# Can the Robson Ten Group Classification System improve the understanding of maternity care in low-income countries? A cross-sectional study in Burkina Faso

**DOI:** 10.1136/bmjopen-2024-086892

**Published:** 2025-03-13

**Authors:** Charles Kabore, Simon Tiendrebeogo, Ana Pilar Betran, Marion Ravit, Michael Robson, Alexandre Dumont

**Affiliations:** 1 Institut de Recherche en Sciences de la Santé, Ouagadougou, Burkina Faso; 2 UNDP-UNFPA-UNICEF-WHO-World Bank Special Programme of Research, Development and Research Training in Human Reproduction (HRP), World Health Organization, Geneva, Switzerland; 3 Université de Paris, IRD, Centre population et développement, F-75006 Paris, France; 4 Obstetrics and Gynecology, National Maternity Hospital and University College Dublin, National University of Ireland, Dublin, Ireland; 5 CEPED, Paris Cité Univesity, Research Institute for Development, Inserm, Paris, France

**Keywords:** Caesarean section, Low-income countries, TGCS, Referral hospital

## Abstract

**Abstract:**

**Background:**

Objective: This study aimed to use the Robson Ten Group Classification System (TGCS) to assess caesarean section (CS) rates and other outcomes in eight referral hospitals in Burkina Faso before the implementation of non-clinical interventions to reduce unnecessary CSs.

**Design:**

This is a cross-sectional study.

**Setting:**

We conducted a 9-month prospective observational study on women who gave birth at eight referral hospitals in Burkina Faso between 1 April 2020 and 31 December 2020.

**Participants:**

We analysed 24 643 women who gave birth at the eight participating hospitals during the study period.

**Outcomes measures:**

We reported the relative size, CS rate and absolute contribution of each Robson group. These indicators were calculated for both referred and non-referred women. Oxytocin administration and stillbirth rates were calculated for women without previous CS and with a single fetus at cephalic presentation at term (groups 1–4).

**Results:**

Overall, 24 643 women gave birth at the eight participating hospitals during the 9- month study period. The overall CS rate was 30.6%. Women in spontaneous labour with a single fetus in the cephalic presentation at term without previous CS (groups 1 and 3) had high CS rates (26.5% and 15%, respectively), low oxytocin use (7.9% and 6.5%, respectively), and high stillbirth rates (3.4% and 3.9%, respectively). These subgroups of women were major contributors to the overall CS rate.

**Conclusion:**

Our results indicate that, in referral hospitals in Burkina Faso, the CS practice for referred women in groups 1 and 3 of the TGCS should receive special attention. These results also reveal areas for clinical improvement to reduce primary CS, especially in nulliparous women. The use of the TGCS is important in low-income countries where low CS rates at the population level may conceal suboptimal labour management in healthcare facilities.

**Trial registeration number:**

ISRCTN67214403.

**STRENGTHS AND LIMITATIONS OF THIS STUDY**

- We evaluated CS practices in a nationally representative sample of referral hospitals in Burkina Faso.
- This study included data on labour management and outcomes (oxytocin use and stillbirths) and stratification according to referral status, allowing a more in-depth interpretation of the findings.
- Our results can be generalised to other referral hospitals in sub-Saharan Africa with similar health systems, where policies for removing user fees have been implemented and may face similar challenges.
- Even if participating hospitals were representative of referral hospitals in Burkina in terms of the level of care, they were located in large cities where most CSs were indicated by obstetricians. Hence, our findings may apply only to this type of healthcare facility.

## Introduction

 The rate of caesarean sections (CSs) has increased significantly worldwide.^1 2^ When medically indicated, CS can effectively prevent maternal and perinatal mortality and morbidity; however, the benefits of increased caesarean deliveries for women who do not need this intervention are controversial. The debate is not as simple as some would make it out to be.^3^,^8^ The increase in CS rates is not limited to high-income settings but affects low- and middle-income countries.^9^ In settings where health systems are under-resourced and have a limited capacity to provide high-quality CSs, an increase in caesarean deliveries may contribute to poorer maternal and perinatal outcomes.^10 11^ In addition, in these settings, the overuse and underuse of CS methods coexist, widening health inequalities.^12^ This approach is particularly critical in low-income countries, where there is an additional burden on already weakened health systems with limited resources.^13 14^ Therefore, CS should be performed only when there is a clear benefit that could offset the higher costs and additional health risks in the specific context of surgery.

Traditionally, monitoring and comparing CS rates have been challenging because of the lack of a standardised, internationally accepted classification system.^15^ In 2015, the WHO stated^2^ that “the priority should not be to reach a specific rate, but to make every effort to practise a CS for all women who need it” and recommended the use of the Ten Group Classification System (TGCS)^16^ as a global standard for the assessment, monitoring and comparison of CS rates in health facilities. This classification system provides a starting point for a more in-depth analysis of the causes and clinical processes of CSs.

### QUALI-DEC project

Since January 2020, the QUALI-DEC (appropriate use of CS through QUALIty DECision-making by health professionals and women) project has been implemented in four countries, including Burkina Faso. The intervention includes four components: (1) opinion leaders who implement evidence-based clinical protocols for CS decision-making; (2) the implementation of the TGCS associated with auditing and providing feedback on CS indications performed for low-risk women; (3) a decision analysis tool to help women make informed decisions on modes of birth and (4) companionship during labour. The QUALI-DEC protocol has been described previously.^17^ In the context of the QUALI-DEC study, the aim of this study was to report the analysis of Robson data collected over a 9 month period before the intervention started and to demonstrate how the TGCS can help providers assess the quality of maternity care in eight referral hospitals in Burkina Faso.

## Materials and methods

### Health system context

The Burkina Faso public healthcare system is pyramidal (Ministère de la Santé du Burkina Faso, 2016) and organised into three levels of care: primary, secondary and tertiary. The first level corresponds to the health district, comprising two sublevels. The first is represented by primary healthcare facilities (CSPSs) and medical centres without surgical units (CMs). The second is district hospitals or medical centres with surgical units (CMAs), which are referral hospitals for health facilities in the district. The second level is represented by the regional hospitals (CHRs), which serve as referral hospitals for CMAs. The third level, the highest level of referrals, comprises university hospitals (CHUs). Basic emergency obstetric care is provided by CMs and CSPSs. To improve access to CSs in Burkina Faso, user fees for CSs have been reduced by 80% in all public hospitals since 2007^18^ and by 100% since April 2016. The fee exemption includes hospital and transport costs for women referred to hospitals. In addition, task-shifting for CS practice has been supported through an additional 3 year training of nurses and midwives as non-physician providers with surgical skills (attachés en chirurgie) and obstetric skills (attachés en gynéco-obstétrique). Nurses with 3 years of additional training in anaesthesia provide most anaesthesia care. Although the national CS rate remains very low (3.7% in 2010–15),^19^ facility-based CS rates steadily increase with unclear medical justification, and most CSs are performed during labour.^20^ Previous studies have questioned whether up to 24% of CSs are medically justified.^20 21^

### Hospitals and participants

We conducted a 9 month prospective observational study on women who gave birth at eight referral hospitals in Burkina Faso between 1 April 2020 and 31 December 2020. These hospitals are included in the QUALI-DEC project and were selected to reflect the context of the healthcare system in Burkina Faso. The eight hospitals are located in urban areas, including three universities, three regional and two district hospitals. Hospital characteristics are presented in table 1. All women who gave birth in participating hospitals during the study period were included.

**Table 1:** Hospital characteristics

Hospital number	Hospital type	Number of deliveries in 2020	Caesarean section n (%)	Instrumental deliveries n (%)
Hospital 1	University hospital	5753	2795 (48.6)	145 (2.5)
Hospital 2	University hospital	4235	1371 (32.4)	28 (0.7)
Hospital 3	Regional university hospital	2905	770 (26.5)	34 (1.2)
Hospital 4	Regional hospital	2919	858 (29.4)	68 (2.3)
Hospital 5	Regional hospital	2089	700 (33.5)	20 (1)
Hospital 6	Regional hospital	3577	1211 (33.8)	25 (0.7)
Hospital 7	District hospital	3834	810 (21.1)	44 (1.1)
Hospital 8	District hospital	5741	905 (15.8)	175 (3.1)

We used the fetal viability threshold at a birth weight ≥1000 g or ≥28 weeks, regardless of the newborn’s condition at birth (alive or dead, with or without malformations) and the delivery route.

Women who delivered at home or another hospital and were subsequently transferred to a participating hospital were not included in this study.

### Definition of core obstetrics variables

The TGCS is based on six variables: (1) parity: nulliparous (women who have never given birth) or multiparous (at least one previous birth); (2) type of gestation: defined as singleton (presence of a single fetus) or multiple (more than one fetus); (3) fetal presentation: cephalic or breech, with the fetus positioned longitudinally, and abnormal positioning, with the fetus positioned transversely or obliquely; (4) previous CS identified by the presence of a uterine scar indicating a previous gestation; (5) pathway to delivery: spontaneous or induced, or pre-labour (when CS was performed before the onset of labour) and (6) gestational age in completed weeks at the time of birth: calculated by the date of the last menstrual period and/or ultrasound (USG) performed up to the 20th week of gestation. However, because gestational age in weeks is poorly documented owing to the low accessibility of early USG, we also recorded gestational age in months, which is usually documented in obstetric records. Midwives were reminded of the need to systematically collect information on women’s gestational age in months when gestational age in weeks was unavailable. When gestational age was recorded in months, a gestational age of at least 9 months was considered equivalent to a gestational age of at least 37 weeks, and a gestational age of less than 9 months was considered equivalent to a gestational age of less than 37 weeks.

Additional data were collected to assess the quality of labour management and facilitate the interpretation of the results. These included the mode of admission (women referred for labour from other healthcare facilities in emergency situations or self-referred women), oxytocin use for acceleration and stillbirths (macerated or fresh) after 28 weeks of gestation in Robson groups 1–4.

### Data collection and management

Based on the six core obstetric variables, the women who gave birth were systematically categorised on a weekly basis into the 10 Robson groups by qualified midwives (data collectors) using the Robson flow chart (online supplemental file 1) and the Robson intermediate table (online supplemental file 2). At the end of the month, the data were consolidated and presented in the monthly Robson classification table (online supplemental file 3). The core obstetric variables were extracted from the women’s medical records and birth registers. The data quality was monitored monthly by the country data manager (ST). Quality control was performed in two steps. First, the data manager compared the number of eligible women reported in the hospital’s birth registers with the number of women classified by the data collector to assess the completeness of data collection and compliance with the eligibility criteria. Second, the data for each woman were reclassified by the data manager and collector together to check for consistency. The validated data were stored by the data manager using the REDCap application.^22^ Additional data, including maternal mode of admission, oxytocin use and stillbirths, were also collected for low-risk women (groups 1–4) and checked on a monthly basis.

### Analysis

The TGCS is a recognised tool that fulfils international and local needs for CS classification.^23^ Based on the six previously defined obstetric characteristics, each woman can be classified into one of ten groups that are totally inclusive and mutually exclusive.^24^ These groups are presented in the WHO Robson Classification Manual.^16^

We first reported the TGCS criteria, which included all women who delivered in the eight participating hospitals during the study period, and then, we stratified the patients according to the mode of admission because we suspected that labour management was different for the women referred and those not referred. The protocol for interpretation of the Robson tables followed the WHO recommendations and steps in the WHO Robson Classification Manual,^16^ and it was synthesised according to the standardised reporting tables provided in the manual. The interpretation of the Robson tables follows a series of steps divided into three main domains: (1) assessment of data quality, (2) assessment of the type of obstetric population and (3) assessment of the CS rates. Data quality assessment focused on group 9. This group includes all women with a single pregnancy with a transverse or oblique lie (including women with previous uterine scars), and the size should not exceed 1%. If this is above 1%, it is probable that women with breech (or other) presentations have been misclassified. Additionally, by convention, the CS rate in this group should be 100%. If the CS rate is not 100%, it suggests that there is probably some misclassification of women in this group (possibly breeches).

The WHO Multi-Country Survey (MCS) was used in both domains for comparison. The WHO MCS is a cross-sectional study implemented in over 300 health facilities in 29 countries and includes over 3 14 000 women from Africa, Asia, the Eastern Mediterranean region and Latin America.^16^ Using data from this survey, a ‘reference population’ was created; this consisted of all the facilities with low CS rates and low intrapartum perinatal mortality. These facilities were assumed to have few unnecessary CSs and good maternal and perinatal outcomes.^16^ The ‘reference population’ comprised 42 637 women from 66 healthcare facilities across 22 countries. The Multi-Country Survey Box presents more detailed information on the WHO MCS and the ‘reference population’.

### Ethics

The QUALI-DEC project was approved by the local and institutional review boards of the Centro Rosarino de Estudios Perinatales of Rosario, Argentina (Record Notice No. 1/20); Pham Ngoc Thach University of Ho Chi Minh City in Vietnam; Khon Kaen University in Thailand; the Ethics Committee for Health Research of Burkina Faso (Decision No. 2020-3-038); the Research Project Review Panel (RP2) of the UNDP/UNFPA/UNICEF/WHO/World Bank Special Programme of Research, Development and Research Training in Human Reproduction (WHO study No. A66006) and the Ethical Review Committee (ERC) of the WHO; and the French Research Institute for Sustainable Development (coordinator of the QUALI-DEC project).

The QUALI-DEC project is registered at ISRCTN67214403, and the protocol has been published elsewhere.^17^ Informed consent was obtained from the local authorities (director of the hospitals and chief of maternity services). Monthly hospital statistics were collected from hospital and medical records. This information and data collection did not require consent from patients in Burkina Faso.

We used the STROBE cross-sectional reporting guidelines.^25^

### Patient and public involvement

No patients were involved in the design, conducting, reporting or dissemination of this study.

## Results

During the study period, there were 24 643 women who gave birth, 13 747 (55.8%) of whom were referred from other facilities. The number of deliveries per hospital ranged from 2089 to 5753. Table 2 shows the Robson classification for all women in the study stratified by referral and non-referral statuses.

**Table 2:** Results of the TGCS, including all the women who delivered at participating hospitals in Burkina Faso from April to December 2020 stratified by referral and non-referral status

	Group	Number of CSs/total deliveries7538/24 643(30.6%)	Size of group*† %	CS rate in group‡† %	Contribution of each group§†	Oxytocin use¶ n/N (%)	Stillbirths¶ n/N (%)
Group 1	**All**	**1771/6677**	**27.1**	**26.5**	**7.2**	**530/6677** (**7.9**)	**226/6677** (**3.4**)
	Referred	1351/4153	30.2	32.5	9.8		
	Non-referred	420/2524	23.2	16.6	3.9		
Group 2	**All**	**307/382**	**1.6**	**80.4**	**1.2**	**19/382** (**5.0**)	**38/382** (**9.9**)
	Referred	129/174	1.3	74.1	0.9		
	Non-referred	178/208	1.9	85.6	1.6		
Group 3	**All**	**1330/8856**	**35.9**	**15**	**5.4**	**578/8856** (**6.5**)	**346/8856** (**3.9**)
	Referred	960/4289	31.2	22.4	7		
	Non-referred	370/4567	41.9	8.1	3.4		
Group 4	**All**	**277/455**	**1.8**	**60.9**	**1.1**	**33/455** (**7.2**)	**57/455** (**12.5**)
	Referred	117/203	1.5	57.6	0.9		
	Non-referred	160/252	2.3	63.5	1.5		
Group 5	**All**	**2165/3638**	**14.8**	**59.5**	**8.8**		
	Referred	1056/1878	13.7	56.2	7.7		
	Non-referred	1109/1760	16.2	63	10.2		
Group 6	**All**	**214/397**	**1.6**	**53.9**	**0.9**		
	Referred	139/271	2	51.3	1		
	Non-referred	75/126	1.2	59.5	0.7		
Group 7	**All**	**260/733**	**3**	**35.5**	**1.1**		
	Referred	145/451	3.3	32.2	1.1		
	Non-referred	115/282	2.6	40.8	1.1		
Group 8	**All**	**465/1374**	**5.6**	**33.8**	**1.9**		
	Referred	307/889	6.5	34.5	2.2		
	Non-referred	158/485	4.5	32.6	1.5		
Group 9	**All**	**192/194**	**0.8**	**99**	**0.8**		
	Referred	137/139	1	98.6	1		
	Non-referred	55/55	0.5	100	0.5		
Group 10	**All**	**519/1819**	**7.4**	**28.5**	**2.1**		
	Referred	333/1225	8.9	27.2	2.4		
	Non-referred	186/594	5.5	31.3	1.7		
Unclassified	**All**	**38/118**	**0.5**	**32.2**	**0.2**		
	Referred	22/75	0.5	29.3	0.2		
	Non-referred	16/43	0.4	37.2	0.1		

Bold numbers are statistics taking into account all women.

* Size of group (%) = number of women in the group/total number of women who delivered in the hospital×100.

† Total numbers of referred and non-referred used for the calculations were 13 747 and 10 896, respectively.

‡ CS rate in group (%) = number of CS in the group/total number of women in the group×100.

§ Contribution of each group=number of CS in the group/total number of women who delivered in the hospital.

¶ Data only available for Ggroups 1, 2, 3, and 4 (all women).

CScaesarean sectionTGCSTen Group Classification System

### Data quality

The step-by-step findings of the data quality assessment are presented in table 3. The total number of CSs and women registered in the REDCap system was similar to those in the participating hospitals during the study period. Overall, 0.8% of the women were in group 9, and 0.5% and 1% of non-referred and referred women, respectively, were in group 9. These rates were consistent with the Robson reference. The CS rate in group 9 was 99%, and the CS rate varied from 98.6% to 100% in referred and non-referred women, respectively, which is consistent with the minimal misclassification of women in this group. The overall percentage of unclassified women was 0.5%, with 0.5% and 0.4% for referred and non-referred women, respectively. This information suggests good data quality.

**Table 3:** Assessment of data quality according to the WHO Robson Classification Implementation Manual^16^

Step forinterpretation	Interpretationaccording to Robson	Example: MCSreference population	Our findings			Interpretation
			Overall	Referred women	Non-referred women	
STEP 1. Total number of CSs	The total number displayed in the table should be identical to the number provided by the official register	NA	Total CS=7538Total deliveries=24 643	Total CS=4696Total deliveries=13 747	Total CS=2842Total deliveries=10 896	There are no missing/incorrect data
STEP 2. Size of group 9	It should be <1%	0.4%	0.8% (n=194)	1% (n=139)	0.5% (n=55)	No evidence of misclassification
STEP 3. CS rate in group 9	By convention, it should be 100%	88.6%	99% (192/194)	98.6% (137/139)	100% (55/55)	The likelihood of misclassification of women with fetuses in the transverse position in this group is very low

CScaesarean sectionMCSMulti-Country Survey

### Types of obstetric population

The size of each group of women is detailed in table 2, and interpretations of these findings are presented in table 4. A total of 66.4% of the women had a single fetus, cephalic presentation at term, and no previous CS (groups 1–4). Around 63% of women were in spontaneous labour (groups 1 and 3). A total of 3.4% of the women had induced labour or pre-labour CS (1.6% in group 2 and 1.8% in group 4). These findings were similar for both the referred and non-referred women. The ratios of the sizes of group 1 versus group 2 and group 3 versus group 4 were much greater than those of both the Robson reference population and the MCS reference population, revealing a low rate of induced labour or pre-labour CS in our hospitals.

**Table 4:** Assessment of the population types according to the WHO Robson Classification Implementation Manual^16^

Step for interpretation	Interpretationby Robson in the WHO Manual	Example: MCS reference population	Our findings			Our interpretation
			Overall	Referred women	Non-referred women	
STEP 1. Size ofgroup 1+group 2	35–42%	38.1%	28.7% (7059)	31.5% (4327)	25.1% (2732)	The combined size of our groups 1 and 2 together is smaller than that of the reference populations. Our population has a high proportion of women who have had more than one child (higher fertility setting).
STEP 2. Size ofgroups 3+4	30%	46.5%	37.7% (9311)	32.7% (4492)	44.2% (4819)	The combined size of our groups 3 and 4 together is greater than that of the Robson reference population but smaller than that of the MCS reference population. This may be explained by a high prevalence of multiparous women in our population.
STEP 3. Size ofgroup 5	Half of totalCS rate	7.2%	14.8% (3638)	13.7% (1878)	16.2% (1760)	The size of our group 5 is in line with the suggested size by the Robson reference, but greater than that of the MCS reference. This suggests that there has been a high CS rate in the past years in these hospitals, and mainly in groups 1 and 2. The large size of group 5 may also explain why multiparous women with a single cephalic pregnancy at term in spontaneous labour or induced labour (groups 3+4) was relatively low (37.7%) as compared with the MCS population (46.5%), although the fertility rate is high in Burkina.
STEP 4. Size ofgroups 6+7	3–4%	2.7%	4.6% (1130)	5.3% (722)	3.8% (408)	The combined size of our groups 6 and 7 together is greater than that of the MCS population but close to that of the Robson reference population. This may be in relation to the high rate of preterm deliveries (size of group 10 over 4–5%).
STEP 5. Size ofgroup 8	1.5–2%	0.9%	5.6% (1374)	6.5% (889)	4.5% (485)	Our group 8 is larger than both the Robson reference population and the MCS reference population. This may be explained by the high prevalence of multiple pregnancies in our population. In addition, the hospitals included in this study are referral hospitals which receive most of the multiple pregnancies.
STEP 6. Size ofgroup 10	<5%	4.2%	7.4% (1819)	8.9% (1225)	5.5% (594)	Our group 10 is greater than both that of the Robson reference population and the MCS reference population. This may also be explained by the fact that the hospitals in this study are referral hospitals which include tertiary hospitals.
STEP 7. Ratio of the size of group 1 vs group 2	≥2	3.3	17.5 (6677/382)	23.9 (4153/174)	12.1 (2524/208)	Our ratio is very high compared with those of both the Robson reference population and the MCS reference population. This could be explained by the low rate of labour induction. Additional data, particularly on the pre-labour stillbirth rate, is needed to understand if these hospitals are not inducing enough women into pregnancy or if the population is very low risk.
STEP 8. Ratio ofsize of group 3vs group 4	>2	6.3	19.5 (8856/455)	21.1 (4289/203)	18.1 (4567/252)	Our ratio is also very high compared with those of both the Robson reference population and the MCS reference population. The same explanation as for the ratio of group 1 vs group 2 could be applied.
STEP 9. Ratio ofsize of group 6vs group 7	Usually, 2	Ratio 0.8	0.5 (397/733)	0.6 (271/451)	0.4 (126/282)	Our ratio is close to that of the MCS population, but lower than that of the Robson reference population. This may be explained by the high number of multiparous women in our population.

MCSMulti-Country Survey

The proportion of women with a previous CS and a single cephalic pregnancy at term (group 5: 14.8%) was in line with that of the Robson reference (half of the total CS rate) but greater than that of the MCS reference population (7.2%), probably reflecting the high prevalence of CS in the past years in these eight facilities, mainly in groups 1 and 2. The large size of group 5 may also explain why the percentage of multiparous women with a single cephalic pregnancy at term (groups 3+4) was relatively low (37.7%) compared with that in the MCS reference population (46.5%); however, the fertility rate is high in Burkina Faso.

Breeches in nulliparous and multiparous women (groups 6+7) represented 4.6% of the entire obstetric population, 5.3% of referred women and 3.8% of non-referred women. This percentage of breeches was higher than that in the MCS reference population (2.7%), which may be explained by the high proportion of preterm deliveries in the participating hospitals. Indeed, the obstetric population included 7.4% preterm cephalic singletons (group 10), 8.9% referred women and 5.5% non-referred women. There were relatively more women with multiple pregnancies (5.6%) than those in the Robson reference (1.5–2%) and the reference population (0.9%). This is related to the fact that in Burkina Faso, multiple pregnancies are managed only in referral hospitals. There may also be a higher incidence of multiple pregnancies in African women. The sizes of groups 6 and 7 were close to those of the MCS reference population but lower than those of the Robson reference population. This finding could also be explained by the high prevalence of multiparous women in the study population.

### Caesarean section rates

The CS rates are detailed in table 2, and their interpretations are presented in table 5. The overall CS rate was 30.6% (34.2% of referred women and 26.1% of non-referred women). Compared with CS rates in both the Robson and MCS reference populations, those in women who underwent spontaneous labour (groups 1 and 3) were extraordinarily high. Consequently, women in groups 1 and 3 were the main contributors to the overall percentage of CSs performed (41% of all CSs). These results were particularly pronounced among referred women (table 2). In these groups, oxytocin use was less than 8%, and stillbirth rates ranged from 3.4% in group 1 to 3.9% in group 3. The very small size of group 2 (1.6%), and its high CS rate (80.4%) and low oxytocin use (5.0%), suggest that group 2 was mainly represented by women with pre-labour CSs. A similar trend was observed in group 4. Stillbirth rates ranged from 9.9% in group 2 to 12.5% in group 4. The data could not be stratified by oxytocin use or stillbirth rate according to the mode of admission.

**Table 5:** Assessment of CS rates according to the WHO Robson Classification Implementation Manual^16^

Steps for interpretation	Interpretationby Robson in the WHO Manual	Example: MCS reference population	Our findings			Our interpretation
			Overall	Referred women	Non-referred women	
STEP 1. CS ratein group 1	Under 10% areachievable	9.8%	26.5% (1771)	32.5% (1351)	16.6% (420)	The group 1 CS rate is higher than that of both the Robson reference population and the MCS reference population, particularly in referred women. The high CS rate in referred women could be explained by the issues in relation to women’s referral in labour, which is a risk factor for CS in this setting, or by suboptimal labour management. The high CS rate in non-referred women could be explained by suboptimal labour and delivery management, including the low use of oxytocin and instrumental delivery. Assessment of caesarean indications in this group is mandatory.
STEP 2. CS ratein group 2	Consistently around20–35%	39.9%	80.4% (307)	74.1% (129)	85.6% (178)	The group 2 CS rate is higher than that of both the Robson reference population and the MCS population. This could be explained by the low incidence of labour induction. Assessment of caesarean indications in this group will be useful for better interpretation.
STEP 3. CS ratein group 3	Not>3.0%.	3.0%	15% (1330)	22.4% (960)	8.1% (370)	The group 3 CS rate is higher than that of both the Robson reference population and the MCS reference population, particularly in referred women. The same explanations as for group 1 could be applied.
STEP 4. CS ratefor group 4	Rarely>15%	23.7%	60.9% (277)	57.6% (117)	63.5% (160)	The group 4 CS rate is higher than that of both the Robson reference population and the MCS reference population. The same explanations as for group 2 could be applied.
STEP 5. CS ratein group 5	Rates of 50–60% are considered appropriate	74.4%	59.5% (2165)	56.2% (1056)	63% (1109)	The group 5 CS rate is lower than that of the MCS reference population but in line with that of the Robson reference population except for non-referred women. The low CS rate in this group may suggest that most of the women had only one previous CS and were eligible for a trial of labour. Women’s referral during labour is an aggravating factor in this setting, and particularly for women with previous CS. Therefore, the assessment of outcomes in this group is crucial to confirm good maternal and perinatal outcomes in relation to the low CS rate.
STEP 6. CS ratefor group 8	Usually, approximately 60%	57.7%	33.8% (465)	34.5% (307)	32.6% (158)	The group 8 CS rate is lower than that of the Robson reference population and the MCS reference population. This could suggest a high prevalence of twin pregnancies with the first twin being in cephalic presentation.
STEP 7. CS ingroup 10	Usually, approximately 30%	25.1%	28.5% (519)	27.2% (333)	31.3% (186)	The group 10 CS rate is higher than that of the MCS reference population but close to that of the Robson reference population. The assessment of CS indications in this group could be useful to understand the high CS rate, particularly to confirm how many were pre-labour or after spontaneous or induced labour.
STEP 8.Relative contribution ofgroups 1, 2 and5 to the overallCS rate	Normally contributeto 2/3 (66%) of all CS performed in most hospitals	Contributed to63.7% of all CS	56.3% (4243)	54% (2536)	60.1% (1707)	CS contributions are lower than both that of the Robson reference population and the MCS reference population. This could be explained on the one hand by the small size of group 2 and on the other hand by the low CS rate in group 5, especially when compared with the MCS population.
STEP 9.Absolute contributionof group 5 tooverall CS rate	NA	Responsible for28.9% of all CS	Absolutecontribution:8.8% (2165/24643)Relative contribution:28.7% (2165/7538)	Absolutecontribution: 7.7% (1056/24643)Relative contribution:22.5% (1056/7538)	Absolutecontribution: 10.2% (1109/24643)Relative contribution:39% (1109/7538)	Absolute contribution is lower than that in the MCS reference population (Robsoncomparison not provided in the WHO manual). This may be in relation to the low CS rate in this group.

CScaesarean sectionMCSMulti-Country Survey

The CS rate in group 5 was lower in our population (59.5%) than in the MCS reference population (74.4%), which is in line with the findings of the Robson reference population. The rate was higher among non-referred women (63.0%) than referred women (56.2%). We suspect that some of the referred for whom a pre-labour CS would have been appropriate were already in labour on arrival at primary healthcare facilities. As a result, they gave birth vaginally at the QUALI-DEC hospitals because they arrived too late for a pre-labour CS. However, group 5 accounted for 29% of all CSs (22% of the CSs in referred women and 39% of those in non-referred women), suggesting that in previous years, the CS rates in groups 1 and 2 were high.

The CS rate among women with multiple pregnancies was relatively low (33.8%) compared with that among both the Robson and MCS reference populations. This suggests that the first twin in twin pregnancies is more likely to exhibit a cephalic presentation. The CS rates among preterm cephalic singletons (group 10) in our population were in line with those among cephalic singletons in the Robson reference population but higher than those in the MCS reference population. This could be explained by the high prevalence of pre-eclampsia or eclampsia before pregnancy at term, which results in preterm pre-labour.^26^

## Discussion

We evaluated the CS practices in eight referral hospitals in Burkina Faso using the TGCS. This approach allowed us to assess the quality of labour and childbirth management in relatively homogeneous groups of women and identify groups with abnormally high CS rates. The results revealed an overall CS rate of 30.6%, ranging from 34.2% in the referred women to 26.1% in the non-referred women. The findings also indicate that in referral hospitals in Burkina Faso, women in groups 1 and 3 undergoing CSs should receive special attention. Verifying the relevance of CS indications in these groups using evidence-based guidelines for CS decision-making is critical. In addition, effective strategies to prevent intrapartum CS, such as labour augmentation, labour companionship and instrumental delivery, are required to reduce potentially unnecessary CSs in these groups.

Compared with women in groups 5–10, women in groups 1 and 3 are less likely to have CS. However, they were the main contributors to the overall CS rate in this study because of the large size of both groups and the relatively high likelihood of these women undergoing CS. These results agree with those of previous studies conducted in low-income countries.^9^ Studies in these settings have identified groups 1, 2 and 5 as the largest contributors to the overall CS rate.^9 27 28^ These findings could be explained by the high fertility rate and a high proportion of multiparous women in low-income countries. However, the very high CS rates in these groups (26.5% in group 1 and 15% in group 3) contrast sharply with the achievable rates (approximately 10% in group 1 and 3% in group 3), warranting more in-depth data collection and subsequent reflection on clinical practices and protocols and subsequent action in these groups.

This is particularly true for women referred from primary healthcare centres to referral hospitals. Our results suggest suboptimal labour management for referred women in groups 1 and 3. This could be explained by the lack of knowledge or use of evidence-based guidelines and the low use of instrumental delivery, as suggested by previous studies in sub-Saharan Africa,^20 29^ which deserves serious consideration. In addition, low oxytocin use may have contributed to the high stillbirth rate in women referred during labour. Indeed, most referred women could have benefited from the augmentation of labour, but this treatment is not allowed in primary healthcare centres in Burkina Faso. In these facilities, only normal delivery can be carried out. Any situation during labour that may require oxytocin use should be referred to a high-level hospital in which oxytocin use is allowed. This is because primary healthcare facilities are not sufficiently equipped and staffed to manage complicated labour. Women in emergency situations are referred during labour from CSPS facilities to referral hospitals and often suffer delays in treatment due to long transportation distances and the need for ambulances, which are unreliable in our setting. In this situation, maternal and perinatal outcomes are jeopardised. A proportion of these women could avoid referral if oxytocin was permitted. In addition to allowing the use of oxytocin in primary healthcare facilities, properly trained personnel should be available to monitor the women. The suboptimal use of oxytocin during labour and emergency referral in this setting are critical risk factors for CS.

Similar to the findings of previous studies in high-, middle- and low-income countries,^9 27 28^ our results showed that having a previous CS (Robson group 5) significantly contributed to the overall CS rate. However, in the present study, the CS rate in group 5 (59.5%) remained lower than that reported in previous studies in low-income countries,^9 30^ and further analysis is needed to confirm good maternal and perinatal outcomes in this group. These results highlight the domino effect of CS use and emphasise the need to ensure the appropriateness of primary CS.^9^

The high proportion of women with multiple pregnancies in our study could be explained by the higher prevalence of multiple pregnancies in sub-Saharan Africa and the fact that only referral hospitals in Burkina Faso are allowed to accept women with multiple pregnancies.

### Strengths and limitations

Our study has several strengths. We evaluated CS practices in a nationally representative sample of referral hospitals in Burkina Faso. Although the QUALI-DEC project took place during the COVID-19 pandemic, the impact on the wards of the QUALI-DEC participating hospitals in Burkina Faso was minimal because the number of cases was very low. This study included data on labour management and outcomes (oxytocin use and stillbirths) and stratification according to referral status, allowing us to provide a more in-depth interpretation of the findings. Our results can be generalised to other referral hospitals in sub-Saharan Africa with similar health systems, where policies for removing user fees have been implemented and may face similar challenges. The potential limitations of this study should be considered when interpreting the results. Even if the participating hospitals were representative of referral hospitals in Burkina in terms of the level of care, they were located in large cities where most CSs were indicated by obstetricians. Hence, our findings may apply only to this type of healthcare facility. TGCS in remote referral hospitals, where most CSs are performed by non-obstetricians, may yield different results.

Although the CS rate at the national level is still very low in Burkina Faso, our results are a manifestation of the wide inequalities in the access to and use of this life-saving procedure in the country, and efforts to reduce unnecessary CS should be considered. Therefore, evidence-based interventions and programmes are required to reduce the incidence of primary CS. Previous studies have shown that a mandatory second opinion on CS indications or the implementation of CS indication audits with feedback helps reduce unnecessary CSs.^31^ The implementation of the latter strategy, combined with the TGCS, could be effective in reducing unnecessary CSs in Burkina Faso.

### Implications for clinical practice

Appropriate pre-service training in evidence-based guidelines for CS decision-making and continuous training in clinical guidelines are important. This is particularly relevant in Burkina Faso, where task shifting has been employed to improve access to CSs. Therefore, although greater access to CSs is a priority in this context, the implementation of evidence-based guidelines for CS decision-making is critical to avoid unnecessary CSs and their associated risks.^31^ In addition, effective strategies to prevent intrapartum CSs, such as labour augmentation, companionship, and instrumental delivery, need to be appropriately implemented.

### Implications for research

Implementation of research is needed to assess the impact of (1) the implementation of evidence-based clinical guidelines for labour and delivery management, including clear indications for the appropriate use of oxytocin and instrumental delivery and (2) the audit and feedback of CS indications in low-risk women on the quality of intrapartum care and CS rates and complications. It is also important to conduct an analysis of antepartum vs intrapartum stillbirths in groups 1 and 3 in relation to potentially insufficient induction (groups 2 and 4). This analysis could inform strategies to improve labour induction and increase pre-labour CS. Finally, an analysis of the outcomes in group 5 to confirm good maternal and perinatal outcomes in relation to the low CS rate in this group is warranted.

## Conclusion

Our results using the TGCS suggest that efforts to improve CS practices and outcomes in referral hospitals in Burkina Faso should focus on reducing unnecessary primary CSs in women who enter labour spontaneously (groups 1 and 3). An in-depth understanding of the use of oxytocin for labour acceleration is urgently needed, not only in referral facilities, but also across the health system, as it could be the cause of higher CS rates. The implementation of evidence-based management of labour and childbirth and an audit of caesarean indications are highly recommended. Methods for fetal and maternal outcome data collection also need to be improved. These findings may be valuable for low-income countries with similar health systems.

## supplementary material

10.1136/bmjopen-2024-086892 online supplemental file 1

10.1136/bmjopen-2024-086892 online supplemental file 2

10.1136/bmjopen-2024-086892 online supplemental file 3

## Data Availability

Data are available upon reasonable request.
