# Supplementary figures and images for: Can the Robson Ten Group Classification System improve the understanding of maternity care in low-income countries? A cross-sectional study in Burkina Faso
Source: BMJ Open. 2025 Mar 13;15(3):e086892. doi: 10.1136/bmjopen-2024-086892 (PMC11907031; doi:10.1136/bmjopen-2024-086892)

**Flow chart for the classification of women in the Robson Classification**


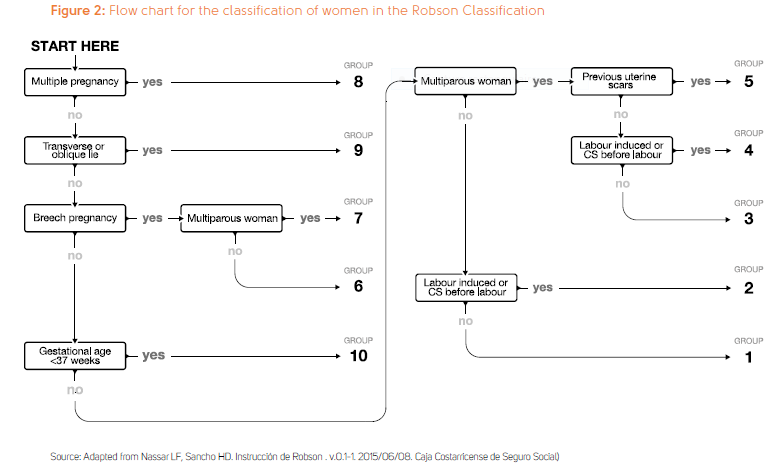

Supplement: online supplemental file 1 [file bmjopen-15-3-s001.docx]
