# Supplementary material for: Can the Robson Ten Group Classification System improve the understanding of maternity care in low-income countries? A cross-sectional study in Burkina Faso
Source: BMJ Open. 2025 Mar 13;15(3):e086892. doi: 10.1136/bmjopen-2024-086892 (PMC11907031; doi:10.1136/bmjopen-2024-086892)
Supplement: online supplemental file 2 [file bmjopen-15-3-s002.docx]

**The intermediate report table to classify 20 consecutive women into the Robson ten-groups**

**Hospital name : ………………………. File number………………………**

**Write inside the table V for vaginal delivery, and C for C-section**

| Robson Group | **1^st^ Case** | 2^nd^ | 3^rd^ | 4^th^ | 5 | 6 | 7 | 8 | 9 | 10 | 11 | 12 | 13 | 14 | 15 | 16 | 17 | 18 | 19 | 20 | **Total**  **V** | **Total**  **C** |
| --- | --- | --- | --- | --- | --- | --- | --- | --- | --- | --- | --- | --- | --- | --- | --- | --- | --- | --- | --- | --- | --- | --- |
| 1 |  |  |  |  |  |  |  |  |  |  |  |  |  |  |  |  |  |  |  |  |  |  |
| 2 |  |  |  |  |  |  |  |  |  |  |  |  |  |  |  |  |  |  |  |  |  |  |
| 3 |  |  |  |  |  |  |  |  |  |  |  |  |  |  |  |  |  |  |  |  |  |  |
| 4 |  |  |  |  |  |  |  |  |  |  |  |  |  |  |  |  |  |  |  |  |  |  |
| 5 |  |  |  |  |  |  |  |  |  |  |  |  |  |  |  |  |  |  |  |  |  |  |
| 6 |  |  |  |  |  |  |  |  |  |  |  |  |  |  |  |  |  |  |  |  |  |  |
| 7 |  |  |  |  |  |  |  |  |  |  |  |  |  |  |  |  |  |  |  |  |  |  |
| 8 |  |  |  |  |  |  |  |  |  |  |  |  |  |  |  |  |  |  |  |  |  |  |
| 9 |  |  |  |  |  |  |  |  |  |  |  |  |  |  |  |  |  |  |  |  |  |  |
| 10 |  |  |  |  |  |  |  |  |  |  |  |  |  |  |  |  |  |  |  |  |  |  |
| Unclassified |  |  |  |  |  |  |  |  |  |  |  |  |  |  |  |  |  |  |  |  |  |  |
| **Sous-Total** | | | | | | | | | | | | | | | | | | | | |  |  |
| **Total** | | | | | | | | | | | | | | | | | | | | |  | |
