# Supplementary material for: Can the Robson Ten Group Classification System improve the understanding of maternity care in low-income countries? A cross-sectional study in Burkina Faso
Source: BMJ Open. 2025 Mar 13;15(3):e086892. doi: 10.1136/bmjopen-2024-086892 (PMC11907031; doi:10.1136/bmjopen-2024-086892)
Supplement: online supplemental file 3 [file bmjopen-15-3-s003.docx]

**The Monthly Report Table**

Hospital (name) ……………………………Month: ………………. Year : …………….

| Robson Group | Number of vaginal deliveries | Number of cesarean deliveries | Total |
| --- | --- | --- | --- |
| 1 |  |  |  |
| 2 |  |  |  |
| 3 |  |  |  |
| 4 |  |  |  |
| 5 |  |  |  |
| 6 |  |  |  |
| 7 |  |  |  |
| 8 |  |  |  |
| 9 |  |  |  |
| 10 |  |  |  |
| Unclassified |  |  |  |
| **Total** |  |  |  |
