# Supplementary material for: Can the Robson Ten Group Classification System improve the understanding of maternity care in low-income countries? A cross-sectional study in Burkina Faso
Source: BMJ Open. 2025 Mar 13;15(3):e086892. doi: 10.1136/bmjopen-2024-086892 (PMC11907031; doi:10.1136/bmjopen-2024-086892)
Supplement: Supplementary file 4 [file bmjopen-2024-086892.reviewer_comments.pdf]

PEER REVIEW HISTORY

BMJ Open publishes all reviews undertaken for accepted manuscripts. Reviewers are asked to complete a checklist review form and are provided with free text boxes to elaborate on their assessment. These free text comments are reproduced below.

ARTICLE DETAILS

Title (Provisional)

Can the Robson Ten Group Classification System (TGCS) improve the understanding of maternity care in low-income countries? A cross-sectional study in Burkina Faso

Authors

KABORE, Charles; Tiendrebeogo, Simon; Betran, Ana Pilar; RAVIT, Marion; Robson, Michael; Dumont, Alexandre

---

VERSION 1 - REVIEW

**Reviewer**	**1**
**Name**	**Akadri, Adebayo**
**Affiliation**	**Babcock University, Obstetrics and gynaecology**
**Date**	**05-May-2024**
**COI**	**There are no competing interests**

I am pleased to read with great interest the Manuscript titled "Can the Robson Ten Group Classification System (TGCS) improve the understanding of maternity care in low-income countries? A cross-sectional study in Burkina Faso"

The content of the manuscript falls within the scope of the Journal. The content will be of great interest to readers.

The study design is appropriate for the study objective.

The presentation of results followed established pattern for reporting ROBSON classification. However, in some instances, the authors dwelled into discussing the results.

The discussion addressed relevant issues.

Overall, the article is interesting and fairly well written. There are however a few concerns that require clarifications:

1. There are two sets of keywords. This has to be harmonized.

2. Introduction

The statement in line 27 : “A standardized internationally accepted classification system for monitoring and comparing CS rates in a consistent and action-oriented manner is lacking because of the lack of factors preventing a better understanding of this trend and its underlying causes (15)”

This statement should be rephrased since ROBSON classification is increasingly being accepted as global standard

3. Table 1

Column 4: The caption: “Caesarean section rate” is not appropriate since the values given are number of caesarean sections (%). I suggest “Caesarean sections n(%)”

Column 5: The caption: “rate of instrumental deliveries is not appropriate since the values given are number of instrumental deliveries (%). I suggest “Instrumental deliveries n (%)”

4. Table 2

The authors should indicate the denominator used to calculate the oxytocin use and still birth rate.

The authors may include a last row showing the relevant total values.

The authors use (n,%), but report as n(%); this should be corrected

The calculation in the caption of the 3rd column is not necessary. The authors can create separate columns for total deliveries per group, and number of CS.

The 30.6% in the caption of the 6th column is unnecessary. It can be at the last row showing totals

The oxytocin use and still birth rates for groups 1 and 3 are incorrect.

5. Caesarean section rate

Line 7: “In these groups, oxytocin use was less than 8%, and stillbirth rates ranged from 3.4% in Group 1 to 3.9% in Group 3”. This should be edited using the correct rates

Line 22:

“We suspected that some of the referred women who benefited from prelabor CSs were in labor at their arrival at primary health care facilities and had vaginal births in the participating hospitals that were not indicated in their records because they arrived late”.

The above statement is unclear and should be rephrased.

6. Discussion

Line 43:

“In addition, in this context, low oxytocin use is associated with a high stillbirth rate in women referred during labor”

Since there was no test of association in this study, the statement needs to be modified. The link between judicious use of oxytocin in labour and good maternal and or foetal outcomes should be made clear.

Line 45:

It is also important to state that: ‘In addition to allowing oxytocin use at PHCs, properly trained personnel should be available to monitor the women….’

7. Delete the “ethics statement” placed before references

**Reviewer**	**2**
**Name**	**Reeve, Nicola F**
**Affiliation**	**Cardiff University**
**Date**	**09-Jul-2024**
**COI**	**No competing interests**

This is an important paper, using the Robson classification to assess the quality of maternity care, with the aim of reducing unnecessary caesarean sections.

The paper has a clear introduction, explaining the background and importance of the study.

The methods section: Data Collection and Management refers to Annex 1-3, which I could not find in the submission. I believe these would help give crucial background information on the Robson classification system to aid understanding for the more general reader - this paper is not accessible to anyone who doesn't already understand (or independently research) the Robson classification system. As this journal is not specific to maternal and neonatal care, I also believe some additional (besides the Annexes) information should be provided fairly early in the methods section (in or before Definition of Core Obstetrics Variables) on what the classification system is and what the groups represent.

The methods section is otherwise clear and comprehensive.

Some of the results section is confusing and hard to follow.

In particular, Table 2:

* it is not clear what the percentages in the referred and non-referred rows are percentages of.

* change 'size of group %' to '% of total deliveries/total referred/non-referred deliveries' or similar.

* 'Contribution of each group 30.6 %' is confusing and misleading - 9.8% is not a contribution to 30.6% but rather the rate of caesareans in all referred patients - which took me too long to work out due to lack of clarity.

* the total referred and non-referred, used for many of these calculations is not given

* Oxytocin and stillbirths - does '-' mean no data? or?

* add total (7538/24643 30.6%) as a separate row, not in header

In the addition to the methods section on the Robson classification system, it would be useful to have a few sentences on the comparisons that are made in the results section (Tables 3 and 4 and associated text). E.g. why does the Data Quality section focus on group 9? The importance/significance of group 9 should be explained in methods. Similarly, a statement on the reasoning behind the comparisons made in Table 4 should be added. A brief description of the MCS reference population should be added, including the expanded abbreviation on first mention – MCS isn't explained anywhere.

The discussion provides an accurate and clear summary of the results, interpretation of results and indications for clinical practice.

I note that the study period covers the worst months of the COVID-19 pandemic. While I appreciate that the numbers of cases in Burkina Faso were relatively low at that time, I wonder if the pandemic had any impact on the results and would be interested to see a statement on this in the discussion.

Minor points:

• Expand abbreviation of CS in abstract, and first use in introduction – abbreviation is never explained.

• Page 4 line 8 +18, and throughout- do you mean neonatal (not perinatal)? If not, it seems unnecessary to include both

• Page 4 line 19 - what approach?

• Page 4 line 30 expand abbreviation of WHO for first use

• Page 7 line 29 - would change 'when the total number of births in the month was reached' to 'at the end of the month'. The former makes it sound like you had a monthly target for the number of births, which I don't think you did?

• Page 9 line 59 - rearrange sentence 'in Group 9, 0.5% and 1%, of the women were nonreferred and referred, respectively' to '0.5% and 1% of the women were in Group 9 and nonreferred and referred, respectively'. In the original, I expect percentages to be of those in Group 9, not the total.

VERSION 1 - AUTHOR RESPONSE

Reviewer #1:

I am pleased to read with great interest the Manuscript titled "Can the Robson Ten Group Classification System (TGCS) improve the understanding of maternity care in low-income countries? A cross-sectional study in Burkina Faso"

The content of the manuscript falls within the scope of the Journal. The content will be of great interest to readers.

The study design is appropriate for the study objective.

The presentation of results followed established pattern for reporting ROBSON classification. However, in some instances, the authors dwelled into discussing the results.

The discussion addressed relevant issues.

Overall, the article is interesting and fairly well written. There are however a few concerns that require clarifications:

Response:

We would want to thanks the **reviewer #1** for this positive comment.

1. There are two sets of keywords. This has to be harmonized.

Response:

We would want to thanks the **reviewer #1** for this relevant comment.

Keywords have been harmonized. Please see page 2 line 16.

2. Introduction

The statement in line 27: "A standardized internationally accepted classification system for monitoring and comparing CS rates in a consistent and action-oriented manner is lacking because of the lack of factors preventing a better understanding of this trend and its underlying causes [15]". This statement should be rephrased since ROBSON classification is increasingly being accepted as global standard.

Response:

We would want to thanks the **reviewer #1** for this relevant comment.

The statement has been rephrased and read now as follow: "Traditionally, monitoring and comparing CS rates had been challenging because of the lack of a standardized internationally accepted classification system [15]".

Please see "Introduction" section, page 3 line 15-16.

3. Table 1

Column 4: The caption: “Caesarean section rate” is not appropriate since the values given are number of caesarean sections (%). I suggest “Caesarean sections n (%)”

Column 5: The caption: “rate of instrumental deliveries is not appropriate since the values given are number of instrumental deliveries (%). I suggest “Instrumental deliveries n (%)”.

Response:

We would want to thanks the **reviewer #1** for this relevant comment.

Columns 4 and 5 of Table 1 have been corrected accordingly.

Please see Table 1 in the “Results” section on page 5.

4. Table 2

The authors should indicate the denominator used to calculate the oxytocin use and still birth rate. The authors may include a last row showing the relevant total values. The authors use (n, %), but report as n(%); this should be corrected.

The oxytocin use and still birth rates for groups 1 and 3 are incorrect.

Response:

We would want to thanks the **reviewer #1** for this relevant comment.

For oxytocin use and stillbirth rates in groups 1 and 3, the numerators were inverted.

We apologize for this error which has been corrected in the revised version of the manuscript. As suggested, the denominators are also shown in the last two columns.

The values are now reported as n/N (%) where n is the numerator and N the denominator.

Please see Table 2 in the “Results” section on pages 8-9.

4. Table 2

The calculation in the caption of the 3rd column is not necessary. The authors can create separate columns for total deliveries per group, and number of CS.

The 30.6% in the caption of the 6th column is unnecessary. It can be at the last row showing totals.

Response:

We would want to thanks the **reviewer #1** for this comment.

Regarding the calculation in the caption of the 3rd column, we followed the international recommendations [16]. WHO recommends that the data is best reported in a standard way using the “Robson Classification Report Table”.

We removed “30.6%” in the caption of the 6th column.

5. Caesarean section rate

Line 7: “In these groups, oxytocin use was less than 8%, and stillbirth rates ranged from 3.4% in Group 1 to 3.9% in Group 3”. This should be edited using the correct rates
Line 22: “We suspected that some of the referred women who benefited from prelabor CSs were in labor at their arrival at primary health care facilities and had vaginal births in the participating hospitals that were not indicated in their records because they arrived late”. The above statement is unclear and should be rephrased.

Response:

We would want to thanks the reviewer #1 for this relevant comment.

Line 7: “In these groups, oxytocin use was less than 8%, and stillbirth rates ranged from 3.4% in Group 1 to 3.9% in Group 3” has been edited with the corrected rates. Please see the subsection “Cesarean section rates” in the “Results” section, page 14 lines 7-8.

The line 22 : “We suspected that some of the referred women who benefited from prelabor CSs were in labor at their arrival at primary health care facilities and had vaginal births in the participating hospitals that were not indicated in their records because they arrived late” has been also rephrased and read now a follow : “We suspect that some of the women who were referred and for whom a pre-labor CS would have been appropriate were already in labor upon arrival at the primary health care facilities. As a result, they gave birth vaginally at the QUALI-DEC hospitals because they arrived too late for a pre-labour CS.” Please see the subsection “Cesarean section rates” in the “Results” section, page 14 lines 15-18.

6. Discussion

Line 43:“In addition, in this context, low oxytocin use is associated with a high stillbirth rate in women referred during labor”

Response:

We would want to thanks the reviewer #1 for this relevant comment.

As suggested, we have edited the text for a more cautious statement. Line 43: “In addition, in this context, low oxytocin use is associated with a high stillbirth rate in women referred during labor” has been corrected and read now as follow: “In addition, in this context, low oxytocin use may contribute to high stillbirth rate in women referred during labor”. Please see the “Discussion” section. Page 17, line 26.

Since there was no test of association in this study, the statement needs to be modified. The link between judicious use of oxytocin in labour and good maternal and or foetal outcomes should be made clear.

Response:

We would want to thanks the reviewer #1 for this relevant comment.

We clarified “The link between judicious use of oxytocin in labour and good maternal and or foetal outcomes” by rephrasing the sentence and it reads now as follow: “Indeed, most of the referred women could have benefited from augmentation of labor, but this treatment is not

allowed in primary health care centers in Burkina Faso". Please see the "Discussion" section page 17 line 27.

Line 45:

It is also important to state that: 'In addition to allowing oxytocin use at PHCs, properly trained personnel should be available to monitor the women….'

Response:

We would want to thanks the reviewer #1 for this relevant comment.

Line 45: We took into account your relevant comment and the sentence read now as follow: "A proportion of these women could avoid referral if oxytocin was permitted. In addition to allowing oxytocin use in primary health care facilities, properly trained personnel should be available to monitor the women" Please see the "Discussion" section. Page 17 lines 32-34.

7. Delete the "ethics statement" placed before references.

Response:

We would want to thanks the reviewer #1 for this comment.

We removed the "ethics statement" placed before references.

Reviewer #2:

This is an important paper, using the Robson classification to assess the quality of maternity care, with the aim of reducing unnecessary caesarean sections. The paper has a clear introduction, explaining the background and importance of the study.

The methods section: Data Collection and Management refers to Annex 1-3, which I could not find in the submission. I believe these would help give crucial background information on the Robson classification system to aid understanding for the more general reader - this paper is not accessible to anyone who doesn't already understand (or independently research) the Robson classification system. As this journal is not specific to maternal and neonatal care, I also believe some additional (besides the Annexes) information should be provided fairly early in the methods section (in or before Definition of Core Obstetrics Variables) on what the classification system is and what the groups represent.

The methods section is otherwise clear and comprehensive.

Response:

We would want to thanks the reviewer #2 for this relevant comment.

Annex 1-3 (supplemental file1-3) have been added to the submission. We also referenced the WHO Robson Classification Manual [16] to help understand what the groups represent.

<u>Some of the results section is confusing and hard to follow.</u>

<u>In particular, Table 2:</u>

<u>* it is not clear what the percentages in the referred and non-referred rows are percentages of.</u>
<u>* change 'size of group %' to '% of total deliveries/total referred/non-referred deliveries' or similar.</u>
<u>* 'Contribution of each group 30.6 %' is confusing and misleading - 9.8% is not a contribution to 30.6% but rather the rate of caesareans in all referred patients - which took me too long to work out due to lack of clarity.</u>

<u>* the total referred and non-referred, used for many of these calculations is not given</u>

<u>* Oxytocin and stillbirths - does '-' mean no data? or?</u>

<u>* add total (7538/24643 30.6%) as a separate row, not in header</u>

Response:

We would want to thanks the reviewer #2 for this relevant comment that pointed out the need of further clarification.

We provided some indications to clarify the results of Table 2. Please see Table 2 in the "Results" section pages 8-9.

Regarding "change 'size of group %' to '% of total deliveries/total referred/non-referred deliveries' or similar", we would like to clarify that we have followed the international recommendations to present the results [16]. WHO recommends that the data is best reported in a standard way using the "Robson Classification Report Table". In this table, data are presented as 'Group Size" and "Group CS rate" in percentage.

- Group size (%) = number of women in the group / total number women delivered in the hospital x 100

- Group CS rate (%) = number of CS in the group / total number of women in the group x 100

We removed 30.6% in 'Contribution of each group 30.6 %' to avoid confusion and misunderstanding.

The total numbers of referred and non-referred, used for the calculations are 13747 and 10896 respectively. We add this information below Table 2.

Yes, the symbol '-' means no data in columns regarding Oxytocin use and stillbirths' rate. For more clarity, we removed this symbol from the columns and we provided some indication to notify missing data regarding Oxytocin use and stillbirths' rate.

We now separated the "total (7538/24643 30.6%)" from the header for more clarity.

Please see Table 2 in the "Results" section pages 8-9.

<u>In the addition to the methods section on the Robson classification system, it would be useful to have a few sentences on the comparisons that are made in the results section (Tables 3 and 4 and associated text). E.g. why does the Data Quality section focus on group 9? The</u>

importance/significance of group 9 should be explained in methods. Similarly, a statement on the reasoning behind the comparisons made in Table 4 should be added.

A brief description of the MCS reference population should be added, including the expanded abbreviation on first mention – MCS isn't explained anywhere.

Response:

We would want to thanks the reviewer #2 for this relevant comment that pointed out the need of further explanation.

We added the following sentences in the "methods section" to explain the comparison that are made in the "results" section and why the Data Quality focus on group 9 including the definition of this group. We also provided a description of MCS reference population.

"The interpretation of the Robson tables follows a series of steps that are divided into three main domains: (i) assessment of data quality; (ii) assessment of type of obstetric population; and (iii) assessment of CS rates. The data quality assessment focused on group 9. Indeed, this group include all women with a single pregnancy with a transverse or oblique lie, (including women with previous uterine scars) and its size should not exceed 1%. If this is above 1%, it is probable that women with breech (or other) presentations have been misclassified. In addition, the cesarean rate in this group should be 100% by convention. If the CS rate is not 100%, this suggests that there is probably some misclassification of women in this group (possibly breeches).

For comparisons, the WHO Multi-Country Survey (MCS) is used in both domains. The WHO MCS is a cross-sectional study implemented in over 300 health facilities in 29 countries and included over 314,000 women from Africa, Asia, Eastern Mediterranean region, and Latin America [16]. Using data from this survey, a "reference population" was created; this consisted of all the facilities with low CS rates and low intra-partum perinatal mortality. These facilities were assumed to have few unnecessary CS and good maternal and perinatal outcomes [16]. The "reference population" included 42,637 women from 66 health facilities in 22 countries. The Multicountry Survey Box presents more detailed information on the WHO MCS and the "reference population"

Please see the "Methods" section, page 7 lines 6-21.

We expanded the abbreviation MCS on first mention.

Please see the "Methods" section. Page 7 line 14

The discussion provides an accurate and clear summary of the results, interpretation of results and indications for clinical practice.

I note that the study period covers the worst months of the COVID-19 pandemic. While I appreciate that the numbers of cases in Burkina Faso were relatively low at that time, I wonder if the pandemic had any impact on the results and would be interested to see a statement on this in the discussion.

Response:

We appreciate the interest of the reviewer #2 and understand the importance of this point in the context in which our study took place. As the reviewer stated, the number of COVID-19 cases was low in Burkina Faso and the measures taken to curb the covid-19 epidemic have had no

significant impact on the operation of maternity wards in hospitals, or on the implementation of the QUALI-DEC project.

We provided the statement on this in the discussion:

"Even though the QUALI-DEC project took place during the COVID-19 pandemic, the impact on the hospital wards of the QUALI-DEC participating hospitals was minimal because the number of cases was low in Burkina Faso"

Please see the "Discussion" section. Page 18 line 12-14.

Minor points:

• Expand abbreviation of CS in abstract, and first use in introduction – abbreviation is never explained.
• Page 4 line 8 +18, and throughout- do you mean neonatal (not perinatal)? If not, it seems unnecessary to include both

• Page 4 line 19 - what approach?

• Page 4 line 30 expand abbreviation of WHO for first use

• Page 7 line 29 - would change 'when the total number of births in the month was reached' to 'at the end of the month'. The former makes it sound like you had a monthly target for the number of births, which I don't think you did?

• Page 9 line 59 - rearrange sentence 'in Group 9, 0.5% and 1%, of the women were nonreferred and referred, respectively' to '0.5% and 1% of the women were in Group 9 and nonreferred and referred, respectively'. In the original, I expect percentages to be of those in Group 9, not the total.

Response:

We would want to thanks the reviewer #2 for this relevant comment that pointed out the need of further explanation.

We expanded abbreviation of CS in abstract, and first use in introduction. Please see line 19 in the "Abstract" section, page 1 line 19 and the "Introduction section" page 3 line 3.

We also expanded the abbreviation of WHO at first use. Please see the "Introduction section" page 3 lines 16-17.

• Page 4 line 8 +18, and throughout- do you mean neonatal (not perinatal)? If not, it seems unnecessary to include both

We mean perinatal death instead of neonatal death.

• Page 7 line 29 - would change 'when the total number of births in the month was reached' to 'at the end of the month'.

We took this into account and the sentence read now as follow: *"*At the end of the month, the data were aggregated and reported on the monthly Robson classification table (supplemental file 3)." Please see the "Data collection and management" subsection. Page 6 line 18.

• Page 9 line 59 - rearrange sentence 'in Group 9, 0.5% and 1%, of the women were nonreferred and referred, respectively' to '0.5% and 1% of the women were in Group 9 and nonreferred and referred, respectively'.

We have rearranged the sentence and it is read now as follow: "Overall, 0.8% of the women were in Group 9, and, 0.5% and 1% of the nonreferred and referred women respectively were in Group 9*"*. Please see the "Data Quality" subsection. Page 9 lines 11-13.

VERSION 2 - REVIEW

**Reviewer**	**2**
**Name**	**Reeve, Nicola F**
**Affiliation**	**Cardiff University**
**Date**	**09-Jan-2025**
**COI**	

I would like to thank the authors for taking the time to revise their manuscript. I am satisfied that they have adequately addressed all comments, with one minor exception:

The abbreviation 'TGCS' is not defined in the main body of the manuscript. It is defined in the title and abstract, but I feel should still be defined on first use in the main body. I believe this to be a minor oversight, as the use of TGCS was changed in the revision process.

Aside of this, the authors have addressed all comments satisfactorily.

VERSION 2 - AUTHOR RESPONSE

Reviewer # 2:

The abbreviation 'TGCS' is not defined in the main body of the manuscript. It is defined in the title and abstract, but I feel should still be defined on first use in the main body. I believe this to be a minor oversight, as the use of TGCS was changed in the revision process.

Response:

We would want to thanks the **reviewer # 2** for this relevant comment.

Now we expanded the abbreviation MCS on first mention in the main body of the manuscript. Please see the “introduction” section, line 17-18 page 3.

Aside of this, the authors have addressed all comments satisfactorily.

Response:

We would want to thanks the **reviewer # 2** for this positive comment.
